# Insights into the Fabrication and Electrochemical Aspects of Paper Microfluidics-Based Biosensor Module

**DOI:** 10.3390/bios13090891

**Published:** 2023-09-19

**Authors:** Rohini Kumari, Akanksha Singh, Uday Pratap Azad, Pranjal Chandra

**Affiliations:** 1 Laboratory of Bio-Physio Sensors and Nanobioengineering, School of Biochemical Engineering, Indian Institute of Technology (BHU) Varanasi, Varanasi 221005, Uttar Pradesh, India; rohinikumari.rs.bce21@itbhu.ac.in (R.K.); akankshasingh.rs.bce22@itbhu.ac.in (A.S.); 2 Laboratory of Nanoelectrochemistry, Department of Chemistry, Guru Ghasidas Vishwavidyalaya (Central University), Bilaspur 495009, Chhattisgarh, India; azadchembhu@yahoo.co.in

**Keywords:** paper-based microfluidics, miniature chips, micro-PADs, fabrications, electrochemical sensors

## Abstract

Over the past ten years, microfluidic paper-based analytical devices (micro-PADs) have attracted a lot of attention as a viable analytical platform. It is expanding as a result of advances in manufacturing processes and device integration. Conventional microfluidics approaches have some drawbacks, including high costs, lengthy evaluation times, complicated fabrication, and the necessity of experienced employees. Hence, it is extremely important to construct a detection system that is quick, affordable, portable, and efficient. Nowadays, micro-PADs are frequently employed, particularly in electrochemical analyses, to replicate the classic standard laboratory experiments on a miniature paper chip. It has benefits like rapid assessment, small sample consumption, quick reaction, accuracy, and multiplex function. The goal of this review is to examine modern paper microfluidics-based electrochemical sensing devices for the detection of macromolecules, small molecules, and cells in a variety of real samples. The design and fabrication of micro-PADs using conventional and the latest techniques have also been discussed in detail. Lastly, the limitations and potential of these analytical platforms are examined in order to shed light on future research.

## 1. Introduction

The concept behind the manufacturing of electronic devices made of paper or other porous materials by controlling very minute quantities of fluids (on the order of 10^−6^ to 10^−9^ L) through them is known as paper-based microfluidics. The capillarity and porosity of paper are utilized in these devices. This concept is becoming increasingly popular due to its undeniable benefits of simplicity, reduced need for laboratory infrastructure, and qualified handling. Also, these devices meet the ASSURED (Affordable, Sensitive, Specific, User-friendly, Rapid and robust, Equipment-free, and Deliverable to end-users) criteria, as established by the World Health Organization (WHO) [1].

Biosensors are the best analytical tools to detect/quantify the electroanalytical or optical response of any electrochemically/optically active biomolecules present in the environment [2]. It is due to their exceptional ability to detect a biological stimulus on a transducing device with a signal proportional to the analyte concentration [3,4,5]. The construction of micro-PADs is being completed for point-of-care (POC) analysis in response to an increased demand for ASSURED diagnostics for infectious, communicable, and non-communicable diseases. In contrast to traditional microfluidic devices that rely on pressure-driven flow like pumps, automated valves, or pneumatic control systems, paper-based microfluidics offer an alternative non-suction flow by creating fluid transport via capillarity. Furthermore, when combined with electrochemical detection methods, these micro-PADs become highly disposable, portable, sensitive, selective, affordable, and only need a modest quantity of materials to perform quick analyses [6]. There are a number of approaches that have been developed to regulate fluid flow in paper-based biosensors, each tailored to a certain functional purpose, such as transport, mixing, or detection [7]. Paper’s low cost, vast availability, and mechanical properties including flexibility, lightness, and low thickness make it an appealing and promising base material for microfluidic applications. The process of pushing a diluted aqueous suspension of cellulose fibers through a screen and drying the resultant sheet produces a sheet made of a network of randomly woven threads. Micro-PADs are being developed using methods that involve cutting hydrophilic channels in paper so that fluids can flow through them by capillary action [8]. Disposable systems utilizing wax as a hydrophobic barrier have seen widespread use for a variety of applications, including the measurement of total proteins, cholesterol, glucose, and harmful drugs in biological fluids [9] and the sensing of heavy metal ions in a water matrix [10]. The substrate of paper is naturally hydrophilic, and in order to contain fluid flow in specific area or guide fluidics along a predetermined path, the creation of a hydrophobic barrier is necessary. These barriers can be produced using a variety of methods, such as photolithography, wax printing, screen printing, plasma treating, flexography, and laser treating [11,12,13,14,15,16]. Standard materials such as silicone, polymers, or glass are widely used in the development of micro-PADs [17,18,19,20,21]. Micro-PADs have additional benefits over conventional microfluidic devices, such as ease of fabrication, portability, biodegradability, low fluid volume processing, cost-effectiveness, minimum sample consumption, and multiplexed assay capabilities [22]. Furthermore, since porous paper naturally acts as a capillary, it does not need pumps or power sources like conventional approaches. A paper channel in a micro-PADs prevents the air bubble that forms in conventional microfluidic channels. Micro-PADs can be easily stored, transported, and scaled up in large numbers at a reasonable price [7]. These features make them a more accessible platform for researchers from a variety of fields [23,24,25,26,27,28]. Micro-PADs have demonstrated significant promise as a robust foundation for clinical diagnosis, food safety, environmental monitoring, and a customized POC sensing platform. The Whiteside group first proposed this idea in 2007 [29], and they have created microfluidic devices by using photoresist-patterned paper to enable liquid movement via capillary force in the absence of any other apparatus.

**Figure 1:**
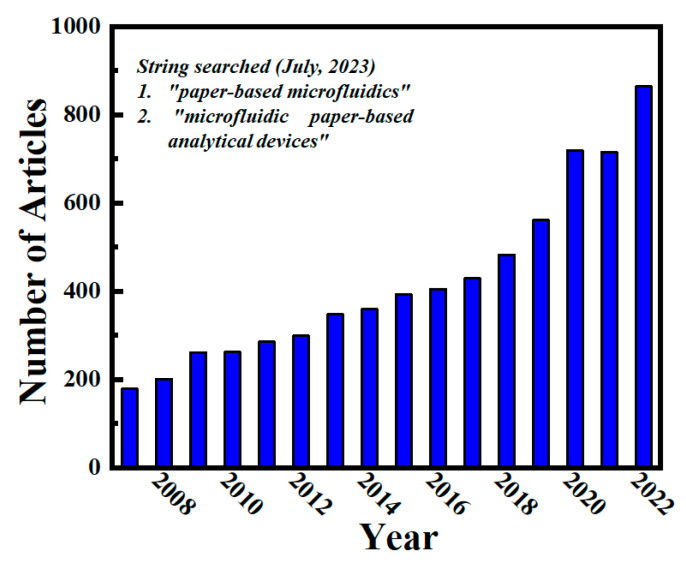
Scientific survey conducted through the online database “PubMed” from 2007 to 2022 using the keywords “microfluidic paper-based analytical device” or “paper-based microfluidics.”.

Because of its potential use as a multiplexable POC platform, micro-PADs are able to execute laboratory activities on a micro-scale for the simultaneous detection of several analytes [30]. When used in fields such as medical, healthcare, and environment, micro-PADs can facilitate the handling and quantitative analysis of fluids [31]. There have been several recent advancements in the domain of miniaturized portable medical tools, which are the result of combining various fabrication techniques with practical diagnostic equipment. Publication trends in this domain show that paper microfluidics is growing at an exponential rate due to its widespread interest. A total of 3321 publications were published between 2007 and 2020; however, if we look at data up to 2022, that number has increased to 6763 papers on the topics of “paper-based microfluidics” and “microfluidic paper-based analytical devices,” respectively, in the PubMed database, as depicted in Figure 1.

Micro-PAD-based electrochemical platforms have a lot of room for improvement, with promising avenues including the creation of novel paper-based systems, the enhancement of system performance with a novel biocatalyst, and the integration of the electrochemical sensor system with other state-of-the-art tools like machine learning and 3D printing.

We have tried to cover recent paper-based microfluidic devices in this review article, particularly electrochemical micro-PADs, which have shown a lot of promise in terms of increased sensitivity and expanded applicability. The development of micro-PADs utilizing both conventional and contemporary techniques has been thoroughly discussed. Also, this review offers a brief overview of the positioning of micro-PADs-based electrochemical platforms and how they are being used now for the quantification of biomolecules (small molecules and macromolecules) and cells in numerous biological fluids and real samples, along with some thoughtful discussion on their applicability in the biotechnology, biomedical sciences and several other domains.

## 2. Designing and Fabrication of Paper Microfluidics-Based Biosensor Module

Owing to the significance of micro-PADs in several fields, especially microfluidic paper-based electrochemical sensors, it is essential to have knowledge of the design and patterning of the microfluidic channel on paper. As already discussed earlier, the construction of these devices involves defining hydrophilic channels and zones on paper by patterning it with hydrophobic barriers. The pioneer work on paper patterning was completed using photolithography on chromatography paper. Several innovative techniques have been evolved day-by-day for the fabrication of advanced micro-PADs. Still, there is a need for more simple, inexpensive, and easy-to-handle fabrication techniques [32]. Two basic steps are involved in the fabrication of micro-PADs. The first is patterning paper, and the second is modification of the devices for their intended use, such as coating the devices with certain reagents. Designing and patterning are performed using computers with certain software, such as AutoCAD, Clewin, CorelDRAW, Illustrator, etc. Recently, researchers have also created a software named AutoPAD with the sole intended purpose of fabricating micro-PADs [7].

**Figure 2:**
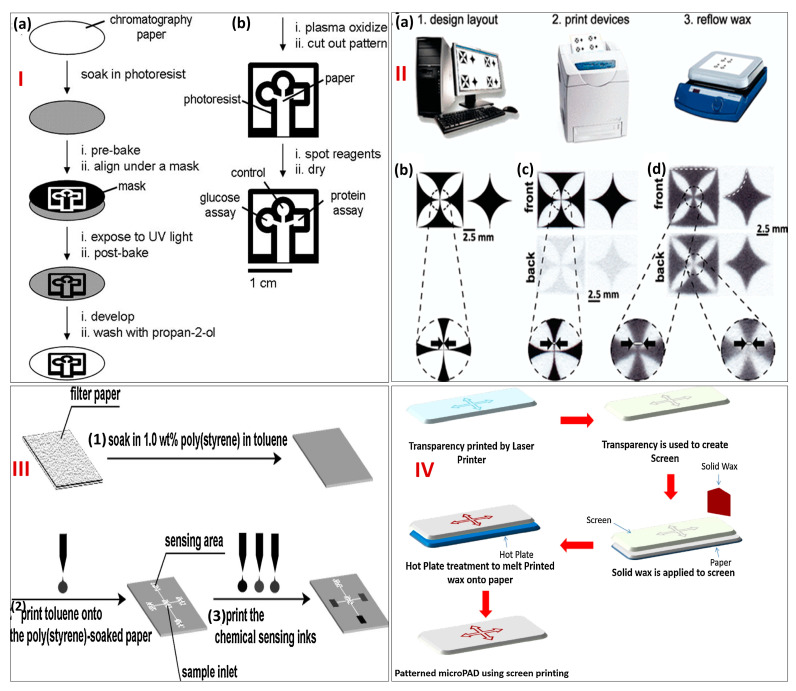
(**I**) (**a**) Schematic representation of step−wise fabrication of a paper−based microfluidic device using photolithography and (**b**) Following photolithography, patterned paper was oxidized using oxygen plasma to make the surface more hydrophilic. Reprinted with permission from [29]. (**II**) Steps involved in the fabrication of paper−based microfluidic devices using wax printing (**a**) depicts the design of the layout on the monitor, printing device, and wax reflow machine, (**b**–**d**) represents the design layout printed via wax printer on the paper. Reprinted with permission from [9]. (**III**) Schematic representation of step−wise fabrication of paper−based microfluidic devices using inkjet printing. Reprinted with permission from [37]. (**IV**) Representation of the fabrication of paper−based microfluidic devices using screen printing. Reprinted with permission from [7].

Some of the conventional and modern design and fabrication techniques have been described here. The first conventional technique for creating micro-PADs is photolithography. It was the first technique to be ever reported by the Whitesides group for paper patterning using chromatography paper. This approach to date stands out for its high level of precision and resolution. It involves transferring a design/pattern to a substrate (like paper) using light. Spin coating is used to put a photoresist (a polymer) solution on the substrate. That is why it is also known as a masking technique. One commonly used photoresist is “SU-8”, which is highly resistive to organic solvents and surfactant solutions [33]. The photoresist-coated substrate is then heated (also known as “soft baking,” “pre baking,” or “post application baking”) to drive off extra solvent. The excess photoresist is subsequently removed from the substrate by submerging it in a “developer,” which exposes the photoresist to a pattern of intense light (usually UV) and a post-exposure bake at 120 to 180 °C. When exposed, a positive photoresist dissolves in the developer, while a negative photoresist becomes insoluble. To solidify the leftover photoresist, the substrate is then baked once again (or “hard-baked”). Post paper pattering, the paper surface is oxidized using oxygen plasma in order to make the surface more hydrophilic. The barrier created has a width of approximately 248 μm and a channel width of 186 μm. Patterned paper can be diversified for different biomedical assays by changing the reagent in the test area. This technique has been used for the fabrication of micro-PADs by several researchers for the detection of analytes such as glucose and proteins [29,33,34]. Another modified photolithography technique is fast lithographic activation of sheets (FLASH) by Martinez et al. in 2008 [35]. Although it is based on photolithography, it simply needs a UV lamp and a hot plate; no cleanroom or specialized equipment is needed. If a UV lamp and hot plate are not available, FLASH patterning can even be completed outside in the sunlight. The technique allows for channels in paper with a width of about 200 μm and a height of about 70 μm with the height depending on the material’s thickness. The biggest drawback of this technique is its cost, as it requires expensive equipment and consumables, and the fabrication is complicated. Figure 2I(a) depicts a schematic representation of step-wise fabrication using photolithography, while Figure 2I(b) depicts the oxidation of patterned paper using oxygen plasma.

Similarly, another one of the most widely used conventional techniques is printing. There are several types of printing methods, but the most popular one is wax-printing. This method is superior due to its simplicity, high resolution, mass production suitability, and lower time consumption. Solid ink printers are widely used, which imprint molten wax rather than ink or toner on the surface of the paper. Fabrication is completed in two steps: (1) printing wax patterns onto a paper surface and (2) melting wax for the formation of a hydrophobic barrier. Heating paper with a wax design printed on one face causes the wax to reflow, allowing it to expand laterally and vertically while passing through the thickness of the paper. The created barrier has a width of about 467 μm and a channel width near about 561 μm. The printing resolution is adequate for the majority of applications, but there are two ways to enhance it. The first technique involves printing on the paper’s faces (front and back with wax). Wax is promptly melted using hot lamination. In the second technique, the paper is shrunk by soaking it in sodium periodate (NaIO_4_) (aqueous), which miniaturizes the patterns. Both of these methods increase the wax printing complexity and can only be used in specific situations. The biggest drawback of this technique is the non-production of solid ink printers and the unnecessary spreading of wax. Therefore, wax printing has been discontinued since 2016. Also, the hydrophobic barriers can be penetrated easily by organic solvents, and the low surface tension of solutions leads to resolution decrement. Several micro-PADs have been fabricated using wax printing [9,36]. Figure 2II depicts a schematic representation of the steps involved in wax printing.

Apart from wax printing, there are several other types of printing also used explicitly for the fabrication of micro-PADs. One such printing method is inkjet printing (Figure 2III). In this printing method, a solvent is used to pattern paper using a commercial inkjet printer. It can be made hydrophobic by immersing it in a polystyrene solution. Following inkjet printing, a preset pattern for the selective removal of some of the polystyrene from the paper is used [36,37]. The advantages of this method are its low cost, quick prototyping, and simple handling. The major disadvantage is the solvent treatment of paper, which is often flammable and clogs printers, thereby hampering the precision of the micro-PADs. Also, this method deploys multiple-layer printing, which decreases the printing resolution. This method has been used for the fabrication of micro-PADs for the detection of glucose, proteins, and pH [38]. Another method is flexographic printing, which uses a sequence of print units to print patterns onto paper using hydrophobic inks such as polystyrene in toluene solutions [39]. The advantage of this technique is that it is less time-consuming and suitable for mass production, whereas the only drawback is the paper surface’s smoothness, which affects printing quality. Similarly, there is the screen-printing method, which uses photolithography to pattern the desired design on a screen (Figure 2IV). Through the patterned screen, the required pattern is created on paper by pressing hydrophobic wax or ink [40]. The greatest advantage of this method is its low cost, while the biggest drawbacks are using a new screen every time for the fabrication and low resolution. Furthermore, one of the recently used methods is laser printing. This method involves printing toner on one side of the paper and then heating the paper to melt some toner components and disperse them throughout the thickness of the paper, which is in contrast to wax printing. A CO_2_ laser is used for cutting, and it is a type of technique in which the pores of paper are blocked physically. The barriers are impermeable to specific surfactants and organic solvents. Laser printers are more accessible and less expensive than solid ink printers, providing good resolution; hence, they are widely used for fabricating micro-PADs [41] for the detection of diseases such as tuberculosis [42].

Apart from the techniques discussed above, one of the simplest techniques of patterning micro-PADs involves paper cutting. In this fabrication method, paper is cut with a laser (CO_2_ laser) or programmable knife cutter into a network of microfluidic channels and zones. To create simple devices, paper might even be manually cut using a knife or pair of scissors. The key benefits of cutting are that it does not require the use of chemicals and only requires a razor blade or a pair of scissors, in its most basic form. The devices are more difficult to control, since the channels are cut out, and they often need strong support to make the channels mechanically stable. The created barrier has a width near about 60 μm, and the channel width is 100 μm. It has been used extensively in the fabrication of reported micro-PADs [43].

**Figure 3:**
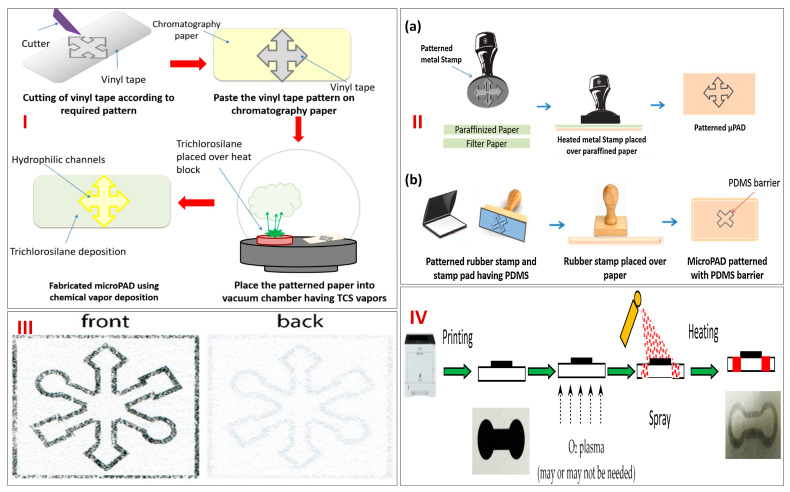
(**I**) Schematic representation of step−wise fabrication of micro−PADs using CVD. Reprinted with permission from [7]. (**II**) Representation of the fabrication of micro−PADs using (**a**) Metal stamping and (**b**) Rubber stamping. Reprinted with permission from [7]. (**III**) Micro−PAD developed using thermal transfer printing. Reprinted with permission from [52]. (**IV**) Schematics of spray on paper printing process for the fabrication of micro−PADs. Reprinted with permission from [53].

There are other techniques also which can be classified under conventional design and fabrication techniques, such as chemical vapor deposition (CVD), vapor phase polymer deposition [44,45,46], plasma treatments [47], and stamps. In work conducted by Lam et al. in 2017 [48], hydrophobic compounds were applied to paper through a mask using CVD. In one variation of this approach, the monomer of compounds like dichloro-[2,2]-paracyclophane (hydrophobic polymer) was evaporated to create a radical monomer, which was then deposited and polymerized on the exposed portions of the paper. This metal mask had the necessary pattern of channels and zones. Another variation of this technique involved cutting a piece of polyvinyl tape into the required pattern and sticking it to paper, which served as the mask. The paper’s exposed regions were subsequently rendered hydrophobic using CVD in a vacuum environment using trichlorosilane. CVD is a type of masking technique like photolithography. Figure 3I clearly depicts fabrication using CVD. Apart from this, for patterning paper, several stamping techniques have been devised. The construction of the stamp and the hydrophobic ink utilized vary a lot between different types of stamping. Depending on the type of ink used, techniques can be divided into two groups: (1) stamping on patterned paper using a hot stamp and liquid inks that dry or cure after being applied to the paper and (2) stamping on patterned paper with molten waxes or polymers, which eventually solidify after cooling. In general, the benefits of stamping technologies are their quick fabrication, low cost of stamps, and modest resource and equipment requirements. In contrast, the major drawback of stamping is the need to fabricate a new stamp for each pattern every time. In order to achieve hydrophobic barriers in paper without overly blurring the pattern, careful optimization of the stamping process and ink is required, as most stamping techniques lack the resolution and precision of other fabrication methods. Several works using rubber stamping and metallic stamping have been reported in the literature [49,50,51]. Figure 3II depicts fabrication using (a) metallic stamping and (b) rubber stamping techniques.

Apart from the previously discussed conventional design and fabrication techniques of micro-PADs, we also have several advanced techniques using novel technology, such as thermal transfer printing and spray printing. These new techniques have evolved in order to remove the drawbacks of the conventional methods. In place of wax printing, thermal transfer printing provides a fresh option for creating micro-PADs. The employed thermal transfer printer is able to manufacture devices that perform on par with those made using wax printing and laser printing in a short period of time [52]. Wax is melted inside specialized printer heads to produce thermal transfer printing. A stationary print head, a carbon ribbon (the ink), and a substrate (typically paper, synthetics, card, or textile materials) are the three essential parts of the thermal-transfer print process. The ribbon resides in the center of a sandwich made up of these three elements. A high-quality printed image is produced by combining the thermally compliant print head with the electrical characteristics of the ribbon and the right rheological characteristics of the ribbon ink. The advantageous output of this type of printing is a high-quality and long-lasting image. However, the ribbons are only intended for single use, which limits this advanced method. Figure 3III depicts paper printed using thermal transfer printing. Similarly, another advanced technique of fabrication is spray on printed paper [53]. In this technique, printing paper is used as a substrate instead of filter paper. The hydrophobic design is created after spraying and heating the toner, which is meant to act as the mask. A systematic examination of processing variables includes toner coverage on the printing paper, hydrophobic spray characteristics, paper surface characteristics, and curing temperature and time. It was discovered that the toner was able to stop the hydrophobic spray (a combination of polydimethylsiloxane (PDMS) and ethyl acetate) from wicking through the printing paper after being printed repeatedly four times. The method is theoretically scalable, and the total processing time for the manufacturing of paper-based microfluidic chips is less than 10 min. Figure 3IV depicts a schematic representation of the spray on paper printing technique.

Various additional fabrication approaches can effectively be discovered to overcome the drawbacks of already existing fabrication techniques. This can be accomplished by focusing on the creation of techniques which are cost-effective, provide high resolution, and involve simple processing steps. Whichever paper patterning approach is developed in the future, it must define hydrophilic channels and zones in a piece of paper by forming precisely defined patterns of hydrophobic barriers. Post patterning and fabrication of micro-PADs, the paper device can further be modified for electrochemical or colorimetric detection. For electrochemical detection, electrodes can be additionally printed using carbon or certain metals. The techniques used for carbon electrode fabrication are screen printing, stencil printing, pencil drawing, and painting, whereas for metal electrodes, the techniques used are thin films, microelectrodes, nanoparticle modification, and wires [54].

## 3. Applications

### 3.1. Paper-Based Microfluidics Platforms for Electrochemical Detection of Small Molecules

Small molecules are organic compounds with low molecular mass (≤1000 Da) and include lipids, secondary messengers, monosaccharides, medicines, metabolites, and other xenobiotics [55]. They can be utilized as research tools to examine biological function as well as for the detection of any harmful clinical molecules. The method that is most frequently employed for the identification and quantification of small molecules is high-performance liquid chromatography with UV and/or fluorescence detection. However, these methods have a number of flaws, including high costs, lengthy processing times, and complicated procedures. Therefore, the sensitive, precise, and rapid identification of small molecules is a challenging task. In the last few decades, paper microfluidic-based sensing systems for the identification of tiny analytes have progressed quickly, and they appear to meet the needs for prompt and sensitive detection. This section discusses several paper microfluidics-based electrochemical sensors for the detection of small molecules.

**Figure 4:**
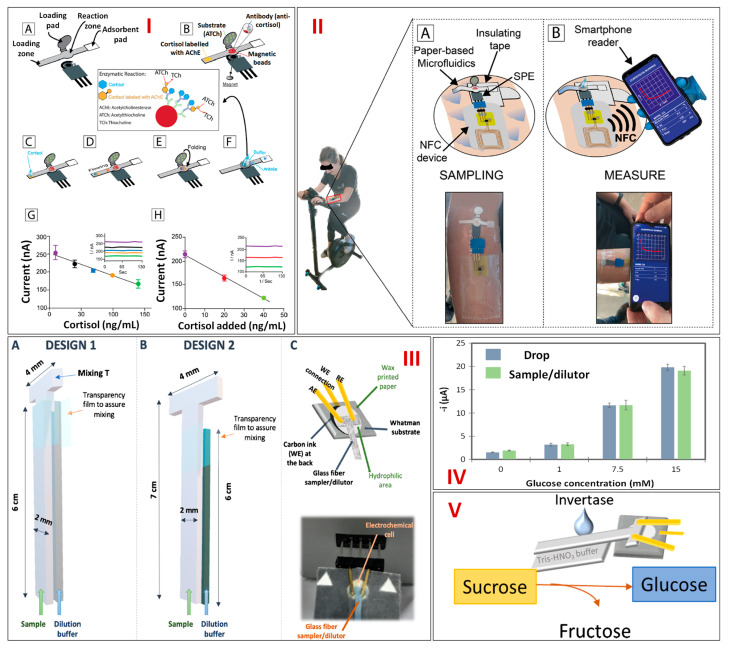
(**I**) Illustration showing the paper microfluidic−based wearable sensor for cortisol sensing in sweat (**A**) depicts the SPE and paper−based microfluidic with its different zones, (**B**) depicts the paper−based microfluidic functionalized with antibodies and different reagents, (**C**) cortisol addition in the loading zone, (**D**) cortisol and cortisol−AChE flowing towards the reaction zone, (**E**) folding of the loading paper, (**F**) addition of buffer to start the enzymatic reaction (inset shows generation of the electroactive thiocholine via the enzymatic reaction), (**G**) calibration curve obtained via microfluidic device (inset shows the corresponding amperometric curves), (**H**) calibration curve obtained in sweat samples with and without spiking cortisol (inset shows the corresponding amperometric curves). (**II**) Schematic representing the cortisol tracking of an individual during cycling by a smartphone−based device. The steps include (**A**) sampling and (**B**) monitoring data via NFC on a smartphone. Reprinted with permission from [56]. (**III**) Schematic representing the two designs constructed for paper–based analytical sensing: (**A**) design 1, which includes one short “T” piece for blending and two straight strips, (**B**) design 2, a straight strip for dilution and a long “T” −shaped sampler, (**C**) Schematic (above) and image (below) of the assembled device. (**IV**) A bar diagram representing the current intensities of glucose concentrations when directly deposited (blue bars) and 20−times diluted samples (green bars) on the electrochemical platform. (**V**) Illustration depicting the enzymatic reaction for sucrose hydrolysis by a paper microfluidics−based electrochemical system. Reprinted with permission from [57].

Cortisol, a glucocorticosteroid hormone, is released by the adrenal gland and regulates several physiochemical factors like glucose levels, blood pressure, and carbohydrate metabolism. Cortisol is specifically responsible for various regulatory processes, including the inhibition of the immune system, the breakdown of fats to provide energy, activation of gluconeogenesis, reducing inflammation, and raising glucose levels by serving as an insulin antagonist. However, elevated amounts of cortisol result in Cushing’s syndrome, which is characterized by signs like tiredness, weight gain, and fragility of the bones, while low levels result in Addison’s disease, which has symptoms including weight loss, tiredness, and wrinkles or scars on the skin. There are several ways to detect cortisol; however, they all need lengthy processes and/or a laboratory setup, which makes it difficult to measure it on-site. To address these issues, current research directions emphasize the creation of POC devices, which can conduct assessments near individuals and offer quick, user-friendly, and easily accessible data pertaining to health. A first paper microfluidics-based system employing filter paper for cortisol testing in sweat was designed by Fiore et al. in 2023. The integrated system was evaluated for its ability to detect cortisol in sweat while cycling and included a microfluidic paper design, magnetic beads (MBs) with magnets, screen-printed electrodes (SPEs), and near-field communication (NFC) boards. First, SPEs were printed using a serigraphic printer onto a transparent and stretchable polyester base, and then, they were altered with Carbon Black/Prussian Blue nanoparticles (CB/PBNPs) (1 mg/mL in N, N dimethylformamide: distilled H_2_O 1:1 *v/v*). Second, solid wax-based printers and filter paper were used to fabricate a paper-based microfluidic design with hydrophilic channels. In order to build a reagent-free system, solutions including 5 μL of MBs, 20 μL of acetylcholine (ATCh), and 20 μL of cortisol–acetylcholine esterase (AChE) were already laden on various locations of the paper-based microfluidic design. The immunological chain was built employing MBs as a tool for the evaluation of cortisol. The accurate measurement of the target analyte was achieved due to the functionalization of monoclonal antibodies on the paper-based microfluidics with the help of MBs (Figure 4I). The paper-based microfluidic pattern’s sample zone was in direct touch with the cycler’s epidermis to collect sweat. Nevertheless, insulating tape was used to keep the SPE and the remaining area of the paper-based microfluidic design away from the cycler’s skin. Mepitel^®^ medical double-sided tape was utilized to secure the NFC board to the skin, and commercially available ties were used to connect NFC to the SPE. The measurement began after around 10 min, when sweat started to moisten the paper-based microfluidics pattern. In detail, after 90 s of sweat entering the reaction area, the pad modified with substrate, ATCh was folded, and then PBS was injected to begin the enzymatic processes by AChE. Following that, the NFC board made electrochemical sensing possible, while the smartphone reader collected data (Figure 4II(A,B)). The recently developed analytical equipment was first tested in standard cortisol solutions, showing a wide linearity between 10 and 140 ng/mL with a limit of detection (LOD) of 3 ng/mL. The effectiveness of this environmentally friendly paper-based integrated device was proven when it accurately measured the amount of cortisol in a person’s sweat during two distinct cycling sessions [56].

Chronic diabetes prevalence is rising quickly, outpacing the capabilities of present healthcare infrastructures and models. By 2030, diabetes is predicted to impact more than 430 million people worldwide, with developing countries accounting for the vast majority of those affected. Micro-PADs, which are constructed using paper as a substrate, have become one of the most promising methods for creating simple, potent, and affordable analytical platforms in recent years. Paper’s porousness is one of the qualities that make it an ideal substrate for microfluidic devices. Gutiérrez et al. in 2021 designed a paper-based microfluidics integrated with electrochemical readouts for the sensitive glucose detection in diluted samples. First, a working electrode made of paper was joined with a three-dimensional microfluidic “dilutor” device made of glass–fiber pads. After that, two designs were prepared and evaluated. The first model (design 1) was made up of three parts: a shorter “T”-shaped strip and two long strips. The “T”‘s stem was positioned in the middle of the two lengthy strips to mix the sample and dilution buffer fluids. The three layers were positioned on top of one another inside a folded transparent sheet that was taped together at the ends to stop leakage. Following that, the “T”-shaped portion outside of the film was kept on the surface of the paper electrode and clamped with a connector header to create an extremely stable platform. The second model (design 2) is a simplified form as it needs two strips, with a “T”-shape end submerged in the sample strip. One of the strips was placed in touch with a dilution buffer, and the other was placed in touch with a solution of sample, and they both were then allowed to move by capillary and interact at the area where the transparency film had been squeezed to enable the blending (Figure 4III(A–C)). In order to show the viability of this platform, the electrochemical cell was altered with the glucose oxidase (GOx), horseradish peroxidase (HRP), and the redox mediator (ferrocyanide) and thereafter combined with sample and dilution strips to measure glucose in various solutions. Enzymatic processes led to the formation of ferricyanide, which was then electrochemically reduced, resulting in the analytical signal. The amount of glucose present has a strong correlation with this current. The current intensities on a calibration plot were identical while measuring glucose directly and after diluting it 20 times (Figure 4IV). Relative standard deviations for all of the observations were below 9.6%, indicating very strong reproducibility. These results indicate the dilutor’s high level of accuracy with reference to the measured dilution factor. Finally, invertase has been applied to the sample strip, and auxiliary enzymatic reactions have been carried out in the same apparatus for the detection of sucrose, which is another sugar that is crucial in determining food quality (Figure 4V). By utilizing the lab-on-paper approach, this technique paves the way for the fabrication of miniaturized devices that can perform decentralized analysis with high precision and can give the same current output in a diluted sample in order to detect glucose within the detection range in a timely manner using cost-effective materials [57].

**Figure 5:**
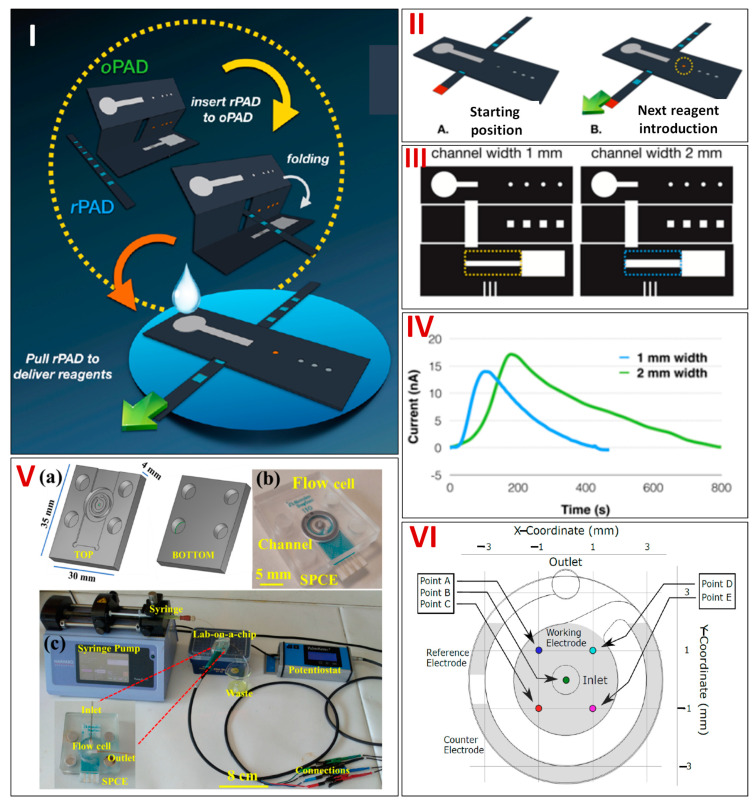
(**I**) Schematic depicting the 3−D sequential paper microfluidic−based electrochemical platform for recognizing ascorbic acid. (**II**) Operation of the analytical device using a flow−through configuration (**A**) depicts the starting position and (**B**) depicts the position where reagent will be introduced. (**III**) The flow−through sePAD is depicted schematically with various channel diameters. (**IV**) Plot illustrating the amperograms of ascorbic acid in relation to channel width. Reprinted with permission from [58]. (**V**) Pictorial representation showing the microfluidic flow cell configuration for detection of glucose (**a**,**b**), Image of the experimental equipment, including the potentiostat, collection vessel, carbon−coated−SPE electrode, and syringe pump (**c**). (**VI**) The position of the flow cell’s working, reference, and counter electrodes is depicted in the image. Reprinted with permission from [59].

Ascorbic acid (AA), one of the most essential small biomolecules, is vital for human physiological function. An excessive amount of AA causes mental disorders, scurvy, cancer, colds, and digestive disorders. Yakoh et al. in 2019 developed an innovative 3D sequential paper microfluidics-based analytical prototype (sePAD) centered on the sliding strip principle (Figure 5I,II). The prototype consists of two major parts: a folded origami paper (oPAD) for electrochemical detection and a mobile reagent storage pad (rPAD) for storing and transferring reagents. This platform, in contrast to the other studies mentioned earlier, permits reagents to be stored within a series of rPAD and carried gradually to the detecting zone of oPAD for continuous flow applications. This enables the creation of a self-calibration plot and a real matrix assessment within one prototype. Using a wax printer, the desired pattern was fabricated on Whatman grade 1 chromatography paper, and then, a wax barrier was made. Furthermore, using a screen-printing approach, three electrodes were fabricated on the rear of the oPAD’s detection area and air dried for 30 min at 65 °C. Three electrodes were attached to a potentiostat using alligator clips. The rPAD’s first hole is specified as an initial position and a direction for the following hole. The underlayer of the second hole of the rPAD is visible from the inlet channel tip’s (layer 1 of oPAD). A carrier buffer arriving from the inlet causes a stored solution to be released, going first to the second hole of the rPAD and subsequently to layer 3 of oPAD. The stored sample solution travels across a detection area after the carrier buffer has been loaded, traversing the first indicator spot on its way to the waste reservoir. When the upward flow from the detection channel causes an orange color to develop in the first place of the indicator region (layer 1 of oPAD), it implies that the resulting solution has been through the detection area and is prepared for electrochemical sensing. The waste reservoir’s bottom was lined with three layers of blotting paper to absorb extra liquid and create a capillary flow of liquid. By altering the width of the channel, it was possible to investigate how device design affected the electrochemical response (Figure 5III, IV). The current increased in a channel with a width of 2 mm; however, a channel with a width of 1 mm was chosen due to its small recovery time and high charge density. A chronoamperogram for several AA concentrations was obtained by introducing a running phosphate buffer solution (PBS); only once and between 0.15 and 0.80 mM, the peak area rose proportionately with increasing AA concentration, with an LOD of 92.8 μM. The sequential microfluidic device provides end users with an affordable and reliable platform, is simple to assemble together, and has the ability to carry out calibration and real sample analysis at the same time [58].

Anemia is a major healthcare concern, particularly for females, and it is the main reason for mortality among mothers in India. One of the most important biomarkers for anemia is ferritin, which is a protein that stores and transports iron. In addition to being a common marker for anemia, it also reveals the extent of oxidative stress. Various fatal disorders, such as Still’s disease, chronic kidney disease, hemophagocytic syndrome, and cardiovascular disease, result from an imbalance in its concentration. The present methods for ferritin quantification typically lack portability or provide a slow response. In an effort to address these problems, Garg et al. in 2020 created a lab-on-a-chip-based sensing platform with a built-in SPE combining microfluidics, electrochemistry, and nanotechnology. The flow cell was designed using AutoDesk Inventor and then built from poly(methyl methacrylate)’s (PMMA) two layers using a computer numerical control (CNC) machine (Figure 5V(a,b)). Graphene oxide with an amine functional group (NH_2_-GO) was added to the carbon-coated SPE surface to enable ferritin antibodies (FerAb) adhesion to the electrode surface. Following that, the FerAb/NH_2_-GO@SPE was placed in the microfluidic flow cell using a straightforward magnetic clamping process to enable continuous ferritin sensing by electrochemical readouts (Figure 5V(c)). The location of the flow cell’s reference, working, and counter electrodes is depicted in Figure 5VI. Due to the immune complex’s insulating properties, the current dropped as the ferritin content rose. The sensor’s linearity range, which is 7.81 to 500 ng/mL, covers the clinically significant concentration band and has a LOD of 0.41 ng/mL. Spiked serum samples were used to test the sensor’s performance. The present method of electrochemical ferritin analysis using microfluidic flow cells showed promising sensitivity and selectivity, and it was proven to be resilient to changes in pH and outside interference. This proved that the proposed technique could potentially be used in POC testing applications [59].

Table 1 comprehensively details the paper-based electrochemical sensors for identifying various small molecules in a real matrix along with their readout method, fabrication strategy, fabrication steps, paper type, detection limits, and dynamic range, which are given below.

### 3.2. Paper-Based Microfluidics Platforms for Electrochemical Detection of Macromolecules

Macromolecules are huge, complex molecules with a high molecular weight (>1000 Da). They are usually made up of monomers, which are covalently linked together. In the biological system, macromolecules play crucial functions in everything from providing morphological support to gaining access to genetic data, activating chemical reactions, and serving as an energy source. This section discusses different paper-based microfluidics-based sensors for the detection of various macromolecules.

**Figure 6:**
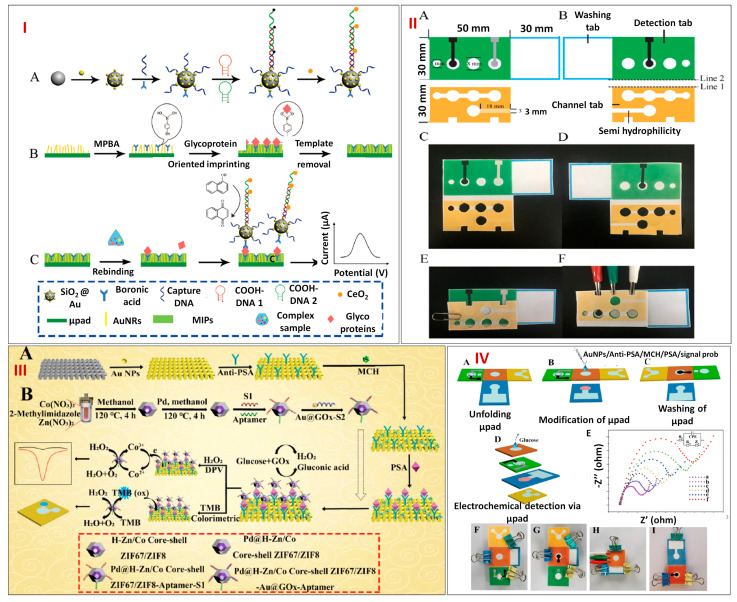
(**I**) A visual representation of the novel strategy based on MIP and sandwich assays for glycoprotein sensing: (**A**) SiO_2_@Au/dsDNA/CeO_2_ signal tag synthesis, (**B**) Boronate affinity−based glycoprotein imprinted film preparation, and (**C**) Stepwise construction of the paper−based interface for indirect OVA sensing. (**II**) Pictorial illustration depicting the size and shape of micro−PADs (**A**,**B**), the development of micro−PADs for OVA detection (**C**,**D**), micro−PADs functioning procedures for (**E**) washing and (**F**) detecting. Reprinted with permission from [73]. (**III**) (**A**) Pictorial representation showing the dual−mode analytical sensing of PSA protein by micro−PADs, (**B**) Stepwise fabrication steps. (**IV**) Schematic showing the assemblage and functioning of the micro−PADs, the steps include (**A**) Unfolding of micro−PAD, (**B**) modification of micro−PAD, (**C**) Washing of micro−PAD, (**D**) electrochemical detection of glucose via micro−PAD, (**E**) EIS plot for each fabrication stage, (**F**–**I**) Image showing the different configurations of micro−PAD. Reprinted with permission from [74].

Glycoproteins are essential for many biological processes, including the regulation of cell growth, cell division, signaling, and its migration. Since the level of glycoproteins is closely linked to the occurrence of disease, numerous glycoproteins have been recognized as indicators for diagnostic purposes. Due to the substantial amounts of co-existing compounds and low concentrations of glycoprotein in the real matrix, excellent selectivity and remarkable sensitivity are required for glycoprotein sensing. A potent method to mimic molecular recognition is molecular imprinting, which has binding characteristics similar to those of an antibody or catalytic activity analogous to those of an enzyme. Sun et al. in 2019 outlined an approach that incorporated hybridization chain reactions and molecularly imprinted polymers (MIPs) into paper microfluidic-based electrochemical sensor for the selective and sensitive sensing of the target glycoprotein ovalbumin, OVA (Figure 6I(A–C)). The wax-printing technology was employed to construct micro-PADs, which were made up of channel, detection, and washing tabs (Figure 6II(A–F)). The working, reference, and counter electrodes were all screen printed on the hydrophilic circle on the detecting tab, which was designated as the sample zone. First of all, Au nanorods with enormous surface areas and excellent conductivity were fabricated on paper cellulosic fiber. The target glycoprotein OVA was then captured using the composite MIPs made up of 4-mercaptophenylboronic acid (MPBA). Thereafter, in the presence of OVA, SiO_2_@Au nanocomposites tagged with MPBA and cerium dioxide (CeO_2_)-modified nicked DNA double-strand polymers (SiO_2_@Au/dsDNA/CeO_2_) were trapped onto the electrode’s surface. Nanoceria were employed as redox-active catalytic amplifiers to generate an electrochemical signal in 1-naphthol’s presence. Therefore, with a comparatively low LOD of 0.87 pg/mL, the developed micro-PAD electrochemical sensor can be employed for indirect OVA sensing in the broad dynamic range between 1 pg/mL and 1000 ng/mL. The OVA recoveries in egg white, which varied between 95.2 and 103.1%, demonstrated that the suggested platform has prospective applications in clinical studies and other relevant domains [73].

Prostate-specific antigen (PSA), another glycoprotein, also acts as a biomarker for various cancers, including prostate, breast, and parathyroid cancer. Modern research has resulted in the emergence of a number of sensitive PSA detection approaches, including electrochemical, electrochemiluminescence, photo-electrochemical, and colorimetric assays. However, it is vital to develop an approach with quick sensing and strong selectivity because of the multitude of tumor markers in human serum. Zhou et al. in 2021 designed dual-mode micro-PADs based on highly electrocatalytic Pd@ hollow Zn/Co core–shell zeolitic imidazolate frameworks, ZIF67/ZIF8 nanoparticles for the sensitive and selective dual-mode detection of PSA. First of all, AuNPs were fabricated on the surface of micro-PADs to amplify the conductivity of the electrode. The Au-micro-PADs interface was then treated with 25 L of 50 µg/mL anti-PSA and incubated at 4 °C overnight in order to immobilize the anti-PSA. Following washing, PBS with varying amounts of PSA was added for interaction with anti-PSA. The modified Au-micro-PADs were then filled with 25 µL of Pd@hollow Zn/Co core–shell ZIF67/ZIF8Au@GOx aptamer, a signal probe, following a unique recognition between the PSA and aptamer. In the meantime, acetic acid and 3,3′5,5′-tetramethylbenzidine (TMB) were inserted in the color region to set up the colorimetric reaction. Then, GOx and glucose were introduced to the detecting region, where glucose quickly underwent an oxidation process and formed H_2_O_2_ (Figure 6III(A,B)). Figure 6IV shows the assemblage and functioning of the micro-PADs along with an electrochemical impedance spectroscopy (EIS) plot for each fabrication stage. At the same time, the signal amplification probe’s Co^2+^ ions quickly oxidized to Co^3+^ ions, and the electron that was liberated contributed to the reduction of H_2_O_2_. Since the concentration of the PSA analyte and the signal probe have a linear relationship, quantitative PSA detection was feasible. As the PSA concentration increased, the DPV current response increased linearly from 1 pg/mL to 50 ng/mL with an LOD of 0.78 pg/mL. The development of hollow metal organic framework (MOF)-based nanocomposites, which could reduce diffusion restriction and give them an ideal shape and sufficient chemical stability, may open up new avenues for enhancing signals in paper microfluidics-based biological evaluation [74].

**Figure 7:**
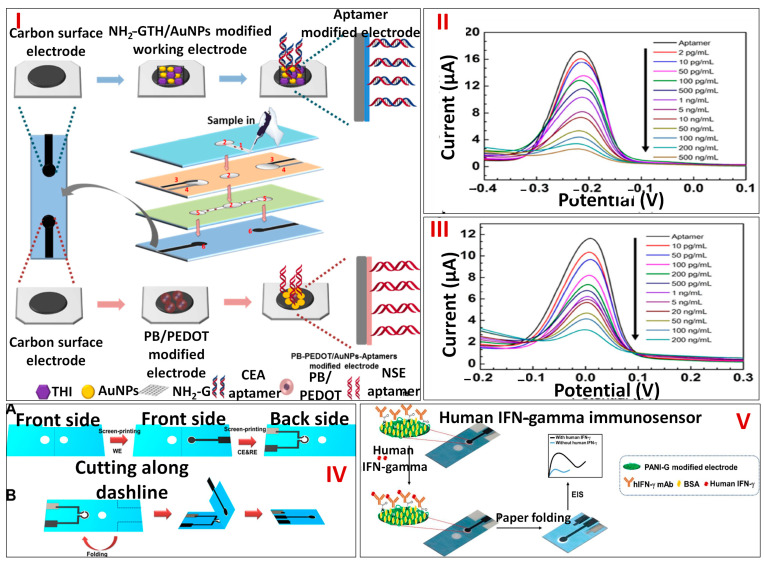
(**I**) Schematic showing the aptasensor–based paper microfluidic device for the detection of NSE and CEA antigens. (**II**) Plot showing the DPV readout for electrochemical sensing of CEA. (**III**) DPV responses to various NSE antigen doses. Reprinted with permission from [75]. (**IV**) Pictorial representation showing the stepwise designing of a paper–microfluidics–based platform for analytical sensing of IFN-γ (**A**) and origami folding (**B**), (**V**) Step-by-step fabrication of human IFN-γ immunosensor. Reprinted with permission from [76].

One of the malignancies that poses the biggest risk to people’s health and lives on a global scale is lung cancer. However, identifying a single tumor marker is of little importance. The rapid and simultaneous detection of several cancer biomarkers with excellent specificity and sensitivity in physiological fluids might assist in diagnosing lung cancer as early as possible. The micro-PADs, a POC technology, have drawn a lot of interest for the multiplex detection of markers. The liquid flow direction of this device could be adjusted to simultaneously detect multiple targets in a single biological sample. Wang et al. in 2019 developed a novel paper-based device for the simultaneous sensing of neuron-specific enolase (NSE) and carcinoembryonic antigen (CEA) markers (Figure 7I). The device, which served as a filter, was made from four cellulose filter papers. First of all, Prussian Blue-poly (3,4-ethylenedioxythiophene)–gold nanoparticles (PEDOT-PB-AuNPs) and amino functional graphene–Thionin-gold nanoparticles (NG-THI-AuNPs) nanocomposites were prepared and fabricated on the working electrode interface. Thereafter, 10 μL of CEA aptamers were introduced on the interface of the NG-THI-AuNPs fabricated electrode, and 10 μL of NSE aptamers was coated onto the PB-PEDOT-AuNPs’ fabricated interface. These steps were then followed by Tris-EDTA buffer rinsing. Due to the integration of two functional electrodes onto a single device, it is capable of concurrently sensing two analytes in a sample. Using the sample inlet on the side, a sample was fed into the device, passed through the microchannel, and eventually arrived at the spot where all three electrodes were screen printed. The basis for identifying the analytes was the reduced response of the DPV, which was caused by the construction of an aptamer–antigen complex on the electrode interface (Figure 7II,III)). The aptasensor demonstrated excellent linearity in the ranges of 0.05–500 ng/mL for NSE and 0.01–500 ng/mL for CEA, respectively. The estimated LOD for CEA and NSE was 2 pg/mL and 10 pg/mL, respectively. The label-free paper microfluidics-based electrochemical aptasensor serves as a novel platform for affordable and early cancer diagnosis particularly in places with limited resources [75].

Human interferon-gamma (IFN-γ), a cytokine that is mainly synthesized by natural killer cells and activated T lymphocytes, is released in response to antigen stimulation. Since the level of IFN-γ rises in response to a particular antigen at the beginning of tuberculosis infection, it is utilized as a biomarker for the disease’s diagnosis. Enzyme-linked immunosorbent assay (ELISA), despite offering great specificity and sensitivity, is laborious and expensive and has a lengthy procedure. As a result, there is a lot of interest in creating an alternative human IFN-γ detection technique for tuberculosis diagnosis that has high sensitivity, affordable prices, rapid interpretation, and disposability. Paper-based electrochemical devices are desirable due to their biocompatibility, flexibility, light weight, high porosity, high sensitivity, and low affordability. Ruecha et al. in 2018 constructed a unique label-free paper microfluidics-based electrochemical immunosensor for screening human IFN-γ. First, the wax-printing approach was employed to engrave the paper-based analytical device with hydrophilic and hydrophobic regions. Three electrode systems were then made by screen printing carbon ink, Ag/AgCl ink, and graphene-modified ink onto the hydrophilic reservoir (Figure 7IV(A, B)). Furthermore, carbon and graphene electrodes were subjected to cyclic voltammetry (CV) measurement for five cycles at a 50 mVs^−1^ scan rate to deposit polyaniline (PANI). In order to covalently immobilize human IFN-γ monoclonal antibodies on the PANI-G altered interface, EDC/NHS was utilized as a coupling agent. The human IFN-γ immunosensor was then exposed with various doses of the protein for 30 min. Following washing, the relative change in impedance was determined using a 5 mM ferri/ferro redox couple [Fe(CN)_6_]^3−/4−^ dissolved in a 0.1 M potassium chloride solution (Figure 7V). The calibration plot showed linearity between human IFN-γ’s logarithmic concentrations and impedance in a range of 5–1000 pg/mL and had a LOD of 3.4 pg/mL. Quick analysis, resilience, simplicity, and disposability are some of the features of this label-free technology. It can therefore be used as a substitute method for detecting human IFN-γ in the early stages of tuberculosis infection [76].

Table 2 comprehensively details the paper-based electrochemical sensors for identifying various macromolecules in a real matrix along with their readout method, fabrication strategy, fabrication steps, paper type, detection limits, and dynamic range, which are given below.

### 3.3. Paper-Based Microfluidics Platforms for Electrochemical Detection of Cells

Paper-based microfluidics devices, besides sensing macromolecules and small molecules, are essential for the identification of dangerous microbes like bacteria and viruses. But the use of paper-based microfluidic devices to identify whole cells has not received much attention. The following section will examine two reported microfluidic paper-based devices that have been developed to detect both whole-cell bacteria and viruses.

**Figure 8:**
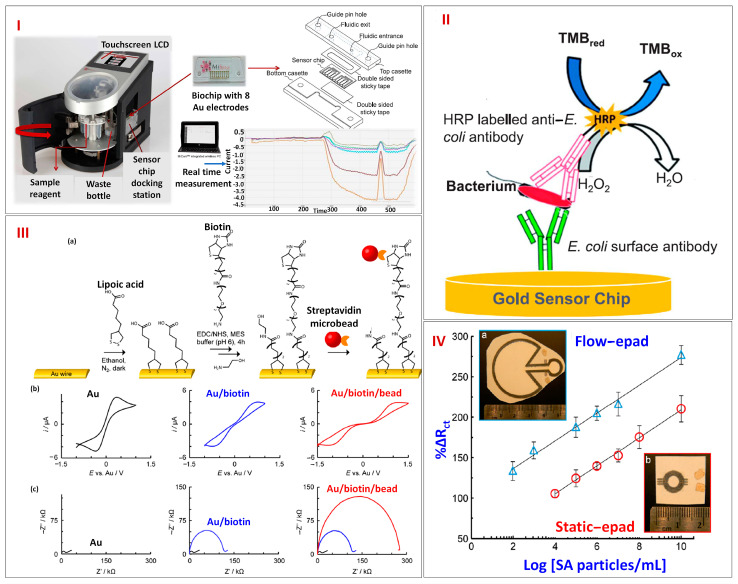
(**I**) Schematic depicting the customized MiSens biosensor for real-time detection of bacteria, (**II**) Screening of pathogens by HRP-labeled secondary antibodies. Reprinted with permission from [91]. (**III**) (**a**) Illustration showing the step-by-step fabrication for the construction of an analytical device, (**b**) Corresponding CV curve for each fabrication step, (**c**) Corresponding EIS plot of each fabrication step. (**IV**) Calibration curve of streptavidin for two different configurations: (**a**) flow-ePADs (blue triangles) and (**b**) static-ePADs (red circles). Reprinted with permission from [93].

One of the biggest health issues around the globe is the presence of bacteria-related waterborne infections. Waterborne illnesses caused by bacteria are among the biggest health issues in the globe. The most widely used techniques for detecting bacteria nowadays include polymerase chain reaction (PCR), cultivation techniques, and approaches that combine these two. There is ongoing interest in developing quick and accurate detection approaches because existing ones are quite laborious and costly. Electrochemical biosensing platforms and microfluidics rank among the most successful forms of detection technologies. Altintas et al. in 2018 designed an automatic microfluidic-based electrochemical device (MiSens) for the detection of bacteria (Figure 8I). The integrated microfluidic apparatus, the novel chip architecture, and real-time amperometric readings are the main characteristics of the MiSens device. The electrode arrays were cleaned using plasma, and a self-assembled monolayer (SAM) was generated by soaking the sensing chips overnight in an ethanolic solution containing 2 mM mercaptoundecanoic acid (MUDA). After being cleaned with water and ethanol, the electrode arrays were dried in a fume hood. Thereafter, the arrays of electrode were sealed in an oxygen-barrier frame and kept at +4 °C until usage. Double-sided adhesive tape was employed to join the PMMA cassettes and SAM-coated electrode arrays. The sensing chip was further placed inside the sensor docking station to provide the fluidic and electronic links necessary to build a channel of microfluidics. Prior to immobilizing antibodies on the sensing areas using traditional amine coupling chemistry, surface characterization using CV was performed. During the intervals between injections, PBS was continuously pumped over the sensor surfaces as the running buffer. Following the initial activation of the sensor surfaces with a 1:1 combination of NHS and EDC, 200 μL of an anti-*E. coli* antibody in sodium acetate buffer was sprayed all over the sensing interfaces. After that, *E. coli* samples were prepared, and each sample was injected onto an electrode surface that had been coated with an antibody. Following this, detecting antibodies conjugated to HRP were introduced. The basic mechanism is an enzymatic reaction occurring in the presence of TMB substrate and HRP. Hence, as the concentration of bacteria increases, the concentration of HRP-labeled detection antibodies rises in the system, resulting in a higher current response (Figure 8II). Using a conventional sandwich assay, *E. coli* was examined in a concentration range varying between 0.99 × 10^4^ and 3.98 × 10^9^ CFU/mL and had a LOD of 1.99 × 10^4^ CFU/mL. The system’s cost might be greatly reduced by being able to regenerate the sensor surface several times. This customized microfluidic sensor has a high specificity and sensitivity for bacteria, making it a promising tool for disease identification [91].

Millions of people are also getting infected by viruses annually in the United States and around the world, significantly raising morbidity and mortality rates in both the developing and industrialized nations [92]. Sensitive detection methods, such as ELISA and reverse transcriptase PCR (RT-PCR), are used to stop the transmission of these pathogens. But these procedures need costly, specialized research centers with highly skilled staff. This points out the urgent need to create an affordable POC testing platform for quick, sensitive, and accurate patient screening. Channon et al. in 2018 constructed a label-free EIS ePAD platform for the sensitive sensing of viral pathogens, including *Streptavidin* (Figure 8III). In this work, two device models are put to the test: a flow-ePAD and a static-ePAD. The initial testing of the device involves the capture of streptavidin-modified nanoparticles by microwires with biotin modifications. The usage of microfluidic platforms over a traditional static paper-based platform improves detection limits because of the improved mass transfer and particle capture on the redesigned electrodes. *Streptavidin* particles were detected by EIS utilizing antibody-functionalized Au microwires. For the trace detection of *Streptavidin* particles with a diameter of 100 nm, the flow-ePAD and static-ePAD platforms were evaluated (Figure 8IV). Although the sensitivities for the two models were similar, flow-ePAD had a higher dynamic range (2.0 × 10^2^ to 2.0 × 10^10^ particles/mL) than static-ePAD, which has a dynamic range of 2.0 × 10^4^ to 2.0 × 10^10^ particles/mL. Hence, flow-ePAD configurations with a low LOD of 7.4 × 10^3^ were chosen for the sensing of viral particles. The ability to specifically detect intact virus pathogens at clinically significant concentrations using this analytical platform is much quicker (30 min) and cheaper ($1 per test) than current approaches [93].

Table 3 comprehensively details the paper-based electrochemical sensors for identifying various cells in a real matrix along with their readout method, fabrication strategy, fabrication steps, paper type, detection limits, and dynamic range, which are given below.

## 4. Potential Applications of Micro-PADs in Various Domains

### 4.1. Healthcare Sector

Micro-PADs are a potential tool for POC diagnosis owing to their simplicity, rapid response rate, portability, and affordability. Some of them have been discussed above for the detection of small molecules, macromolecules, and cells. Other medical applications include plasma separation, blood typing, and the detection of hormones and other biomarkers in invasive and non-invasive body fluids. Blood is a crucial analyte for screening of any kind of disease, pathogen, or deficiency, as it serves as a medium for the transportation of vitamins, minerals, carbohydrates, proteins, lipids, and microorganisms in the body. Serum/plasma separation from blood is important for screening purposes. For instance, a micro-PAD has been devised for the separation of plasma from whole blood for the determination of glucose [96]. Similarly, blood typing is crucial for blood transfusion and organ transplantation. Recently, a simple and affordable micro-PAD has been devised for Rh phenotyping [97]. In addition to this, paper-based microfluidics platforms are widely being used for the detection of pregnancy hormones, human chorionic gonadotropin (hCG) in urine [98] and amyloid beta peptides in various body fluids [99]. Hence, the healthcare sector employs extensive use of micro-PADs.

### 4.2. Environmental Sector

Environmental pollution and degradation have become one of the biggest concerns of the century due to ever-expanding urbanization and population. Heavy metal toxicity is one of the major contributors to the degradation of soil and water. Several micro-PADs have been developed for the detection of toxic metals such as Pb^2+^, Cd^2+^, and Zn^2+^. The first attempt has been made by Nie et al. by developing an SPE crafted out of paper with patterned micro-channels [10]. Recently, an electrochemical biosensor has been crafted of office paper for the detection of paraoxon-ethyl in fertilized soil and normal soil samples [100]. Likewise, efforts have been made to detect diclofenac (DCF) in tap water [101]. Also, paper has been utilized in micro-PADs for the development of energy storage devices [102]. Therefore, micro-PADs have great utilization in the environmental sector.

### 4.3. Food Sector

Food is the most essential component for sustaining life. With an increasing awareness on healthy and quality food, food quality assurance has become a significant aspect in the food industry. It is not only related to the aesthetic appearance of food but also to the microbiological and chemical composition also. To maintain food quality across the supply chain, several micro-PADs have been made using paper. For instance, for the detection of the meat preservative “nitrite”, micro-PADs have been developed [103]. A further modification to this device has been made by improving the LOD value of the device [104]. Similarly, for determining the amylose content of starch in rice (1.5% to 26.4%), a micro-PAD has been developed [105]. There are many more developed devices for food quality assurance, highlighting the utilization of micro-PADs.

## 5. Conclusions and Future Prospects

Paper-based microfluidics, a rapidly expanding domain, has garnered substantial attention and popularity due to its large number of advantages over conventional microfluidics. These include facile construction using well-known patterning techniques, ease of use, flexibility, the ability to regulate fluid flow without machinery, and quick response. Paper is currently fulfilling the ideal base material criteria for the technology transfer of these advanced, novel micro-PADs fabrication protocols. As a result of the superior properties of paper, micro-PADs are developed in response to provide simple and low-cost analytical platforms that are easily manageable by end-users for a wide array of applications. These applications are increasing day by day due to the improved capabilities of micro-PADs in sample preparation, fluid control, and miniaturization. Miniaturization and microfabrication technologies have largely influenced the rapid advancements in biosensors, both in terms of research and product development, for better communication between biological sciences and engineers/physicists. In the recent past, three electrode-based electrochemical sensing systems based on paper-based microfluidics have garnered a lot of interest. Hence, in this review article, we have presented a concise overview of the state-of-the-art micro-PADs fabrication methods and how they are currently being used to electrochemically quantify biomolecules (both small and large molecules) and cells in a variety of real samples. We also engage in a thoughtful discussion of their potential applications in biotechnology, biomedical sciences, and various other domains. Although paper-based microfluidic devices have a wide range of potential applications, there are several challenges that need to be addressed. Sample retention in microfluidic channels, the variability of paper, and the limited shelf-life of enzymes on micro-PADs are some of the biggest drawbacks. Additionally, when the surface tension of the sample is low, it starts to penetrate the hydrophobic channels of wax. High LOD values are also a concern, as they limit the detecting ability of these devices with low concentrations of analytes in them. These limitations can be overcome by using more uniform paper and by integrating advanced nanomaterials into micro-PADs. There are very few commercial products based on conventional microfluidics. Due to the paper’s accessibility and a number of other enticing features, micro-PADs serve as the ideal platform for the transition of micro-PAD-based sensing technology from the lab to the market. Micro-PAD’s appealing qualities that allow for quick tests and prompt results for end-users are what make it commercially feasible. Researchers are working hard to develop wearable sensors based on micro-PAD technology for the commercial market that can track therapeutically important components in bodily fluids, including perspiration, saliva, tears, and bodily fluids like blood. However, in terms of commercialization, paper-based microfluidics still have a long way to go before they can be considered a true POC testing model with the cutting-edge capabilities of analyte purification, multiplexing analysis, quantization, and tracking with high sensitivity and selectivity. Despite the fact that this subject is still in its infancy, it is anticipated to grow quickly and offer solutions to some of the most urgent challenges in modern healthcare. It will be integrated with cutting-edge technologies, including deep learning, artificial intelligence, machine learning, and the Internet of Things, in the coming years for POC applications.

## Figures and Tables

**Table 1:** Paper microfluidics-based electrochemical devices for the detection of small molecules.

	Sensing Molecule	Electrochemical Technique Used	Fabrication Strategy	Fabrication Steps	Paper Type	LDR	LOD	Real Sample	References
1	Cd^2+^, Pb^2+^	SWASV	3D microfluidic paper and graphite foil used to fabricate sensor. Modified working electrode prepared using Bi_2_O_3_.	3	Filter paper (pore size: 12−15 μm; thickness: 305 μm)	5–500 μg/L for Cd^2+^ and 5–100 μg/L for Pb^2+^	1.2 μg/L for Cd^2+^, 1.8 μg/L for Pb^2+^	Commercial mineral water	[60]
2	Paraoxon (Nerve agent stimulant)	CV and CA	Working electrode modified with Carbon Black/Prussian Blue nanocomposite powder. Paper-based test area was integrated with nitrocellulose membrane holding SPCE and BChe.	3	Filter paper (density: 67 g/m^2^)	5–100 μg/L	3 μg/L	NR	[61]
3	Bisphenol A	CV, DPV	MWCNT electrode modified by ZnO.	3	NR	0–5 mM	0.35 μM	NR	[62]
4	Glucose	LSV	SPCE were paired with paper-based gold working electrodes to produce gold nanoparticles electrochemically in a repeatable manner.	8	Whatman chromatographic papers grade 1 (180-μm thick) and grade 3MM (260-μm thick)	0.01–5 mM	6 μM	Orange juice, energetic beverage and cola beverage	[63]
5	Salivary thiocyanate	SWASV	A micropump made from a capillary channel that was created via laser-engraved micropatterning on paper. CuPc/SPGE, a graphene electrode modified with copper (II) phthalocyanine, was used.	3	Filter paper (Whatman No. 1)	0.025–100 mmol/L	6 μmol/L	Real human saliva	[64]
6	Lidocaine	SWV	Integrated graphene-based paper microfluidic electrode. Laser printing and NIR drying utilized for fabrication.	6	Filter paper (Whatman, φ = 55 mm with particle retention of 20–25 μm)	1–100 μM	0.8 μM	Serum and blood	[65]
7	17β-estradiol	CV and DPV	Modified electrode fabricated using amine-functionalized SWCNT/new methylene blue/ (AuNPs).	4	Chromatography paper (Whatman No. 1)	10 pg/mL–500 ng/mL	5 pg/mL	Clinical serum samples	[66]
8	Creatinine	CV and amperometry	Electrochemically reduced graphene modified SPCE (CuO/IL/ERGO/SPCE) on a PAD.	5	Filter paper (Whatman no. 1, Camlab, UK)	0.01–2.0 mM	0.22 mM	Human serum sample	[67]
9	Glucose, lactate, and uric acid	CA	Microfluidic channels designed using photolithography and electrodes made using screen printing.	3	Filter paper (Whatman grade-1)	0–100 nM (Glucose), 0–50 nM (Lactate) and 0–35 nM (Uric acid)	0.21 mM (glucose), 0.36 mM (lactate), 1.38 mM (uric acid)	Human serum sample	[14]
10	Adenosine	CC	Origami paper analytical device with microfluidic channel made using wax printing and electrode fabricated using screen printing with glucose oxidase labeled DNA aptamer.	4	Chromatography paper (Whatman grade-1)	NR	11.8 μM	NR	[68]
11	Paracetamol and 4-amino phenol	CA	Wax printing used to create microfluidic channel and electrochemical detection system made using sputtering at the end of channel.	4	Chromatographic papers n1 and p81 (Whatman)	NR	Paracetamol: 25 μmol/L(paracetamol) and 10 μmol/L (4-amino phenol)	Pharmaceutical tablets	[69]
12	Ascorbic acid and sunset yellow	CA	Wax printing is used to fabricate microfluidic channel and pencil drawing used to create electrode.	3	Filter paper foil type 1 (Whatman)	50–1000 μM	30 μM (ascorbic acid) and 90 μM (sunset yellow)	NR	[70]
13	Melamine	DPV	Electrodes fabricated using ink writing using ball-pen device.	4	A4 paper of 70 mg	0–100.0 μΜ	1.0 μM	Artificial urine sample	[71]
14	Pentachlorophenol	PEC	Combination of micro-PADs and molecular imprinting technique. AuNP-decorated working electrode with polypyrrole-functionalized Zno NP.	6	Chromatography paper No. 1 (Whatman)	0.01–100 ng/mL	4 pg/mL	Spiked samples with river water and pure drinking water	[72]
15	Pb^2+^	ASV	Micro-PADs fabricated by photolithography/wax printing and electrode fabricated using screen-printing.	3	Chromatography paper (Whatman No. 1)	NR	1.0 ppb	NR	[10]

CV—Cyclic voltammetry, MIP—Molecularly imprinted polymer, EIS—Electrochemical impedance spectroscopy, DPV—Differential pulse voltammetry, LSV—Linear sweep voltammetry, CA—Chronoamperometry, GCE—Glassy carbon electrode, SPCE—Screen-printed carbon electrode, SWASV—Square wave anodic stripping voltammetry, BChe—Butyrylcholinesterase, MWCNT—Multi-walled carbon nanotube, HPV—Human papilloma virus, CuPc/SPGE—Copper (II) phthalocyanine-modified screen-printed graphene electrode, NIR—Non-destructive near infrared, CRN—Creatinine, UA—Uric acid, SWV—Square wave voltammetry, SWCNT—Single-walled carbon nanotube, AuNP—Gold nanoparticle, CC—Concentration cell, PEC—Photoelectrochemistry, AuNP—Gold nanoparticle, NP—Nanoparticle, ASV—Anodic stripping voltammetry, NR—Not reported.

**Table 2:** Paper microfluidics-based electrochemical devices for the detection of macromolecules.

	Sensing Molecule	Electrochemical Technique Used	Fabrication Strategy	Fabrication Steps	Paper Type	LDR	LOD	Real Sample	Reference
1	PD-L1	DPV	Paper microfluidic device aptasensor created. Amine-functionalized single-walled carbon nanotube, new methylene blue and gold nanoparticles nanocomposites synthesized for binding aptamer.	3	Chromatography paper (Whatman No. 1)	10 pg/mL– 2.5 ng/mL	10 pg/mL	Serum sample	[77]
2	ALP, miRNA	DPV	Paper modified with signal molecule labeled DNA along with target recognition solutions and screen-printed electrodes.	3	Cellulose chromatography paper (Whatman)	NR	NR	Human serum sample	[78]
3	EGFR	CV and DPV	The working electrode was altered using amino-functionalized graphene (NH_2_-GO), thionine (THI), and gold particle (AuNP) nanocomposites.	3	Chromatography paper (Whatman No. 1)	0.05–200 ng/mL	5 pg/mL	Serum	[79]
4	CEA	DPV	The reference electrode was created using commercial silver ink, the working and counter electrodes were printed using a gold nanoparticle ink.	3	Grade 1 chromatography paper (Whatman^®^)	1.0–100.0 ng/mL	0.33 ng/mL	Serum	[80]
5	HCG	DPV	Antibodies conjugated covalently on hydrophilic detection zone of SPCE. Electrochemical immunofiltration designed and constructed.	4	Chromatography paper (Whatman No. 1)	1.0 mIU/mL–100.0 IU/mL	0.36 mIU/mL	Serum	[81]
6	miRNA-155	EIS	Modified paper-based electrode using gold nanoparticles (AuNP-PE)	5	Nitrocellulose membrane	0–1.5 μg/mL	93.4 nM	Fetal bovine serum	[82]
7	Exosomes	DPV	Paper-based three electrode devices made with parafilm hot film, which was later cut by laser for making patterns.	5	Chromatography filter paper (CHR, Whatman, UK)	10^8^–10^10^ exosomes/mL	9.3 × 10^7^ exosomes/mL	Human serum and plasma	[83]
8	PSA	DPV	Wax-printed and screen-printed electrode modified with AuNPs/rGO/THI nanocomposites for immobilization of DNA aptamer probe.	5	Chromatography paper No. 1 (Whatman) pure cellulose paper	0.05–200 ng/mL	10 pg/mL	Serum	[84]
9	HPV B sAg and HPV C cAg	Chronoamperometry	Wax printing was completed on paper followed by electrode fabrication using carbon-based ink using in-house screening method.	8	Filter paper	0.1–250 ng/mL for HBsAg and 0.001–250 ng/mL for HCVcAg	18.2 pg/mL for HBsAg and 1.19 pg/mL for HCVcAg	Clinical serum samples	[85]
10	Claudin 7 and CD81	Amperometry	Dual electrochemical immunosensor with paper designed by wax printing while electrodes printed using GO and silver ink.	4	Filter paper (Whatman No.1)	2–1000 pg/mL for Claudin 7 and 0.01–10 ng/mL	0. pg/mL for Claudin 7 and 3 pg/mL for CD81	60 breast cancer patients and 20 healthy volunteers	[86]
11	PEAK 1	DPV	Paper-based electrodes immobilized with GO for the incorporation of antibodies.	3	Chromatography paper (Whatman No.1)	10–106 pg/mL	10 pg/mL	NR	[87]
12	miR-21	SWV	Paper modified with signal molecule labelled DNA and electrode made using screen-printing electrode.	3	Cellulose chromatographic paper (Whatman)	1 fM–1 μM	0.1 fM	Serum sample	[78]
13	AFP	DPV	Origami-based electrochemical immunodevice-based PANI-AuNP-modified PWE.	NR	NR	0.001–100 ng/mL	0.80 pg/mL	Human serum	[88]
14	Milk allergen casein	DPV	Electrode made of GN/CN/GelMA composite material and paper microfluidic channels fabricated using wax printing.	5	Pure cellulose paper	1 × 10^−7^–1 × 10^−6^ g/mL	0.032 μg/mL	Rat basophilic leukemia mast cells	[89]
15	CA-125	DPV	Graphene used to modify immunosurface device. AGET ATRP used.	3	Chromatography paper no.1 (Whatman)	0.05–100 ng/mL	0.05 ng/mL	Human CA-125	[90]

PD-L1—Programmed death-ligand 1, DPV—Differential pulse voltammetry, ALP—Alkaline phosphatase, CV—Cyclic voltammetry, MIP—Molecularly imprinted polymer, EIS—Electrochemical impedance spectroscopy, LSV—Linear-sweep voltammetry, CA—Chronoamperometry, GCE—Glassy carbon electrode, GN—Graphene, SPCE—Screen-printed carbon electrode, SWASV—Square wave anodic stripping voltammetry, BChe—Butyrylcholinesterase, MWCNT—Multi-walled carbon nanotube, GO—Graphene oxide, CEA—Carcinoembryonic antigen, SPCE—Screen-printed carbon electrode, SWV—Square wave voltammetry, AuNP PE—Gold nanoparticle paper–electrode, PSA—Prostate-specific antigen, AuNPs/rGO/THI—Gold nanoparticles/Reduced graphene oxide/thionine, HPV B—Human papilloma virus B, HPV C—Human papilloma virus C, cAg—Core antigen, sAg—Surface antigen, PEAK-1—pseudopodium-enriched atypical kinase one, SGK269, AFP—Alpha ferro protein, CA-125—Cancer antigen-125, PANI—Polyaniline, PWE—Primary working electrode, AGET ATRP—Activators generated electron transfer for atom transfer radical polymerization, CN—Carbon nanofiber, GelMA—Gelatin methacryloyl, NR—Not reported.

**Table 3:** Lists of paper microfluidics-based electrochemical devices for the detection of cells.

	Sensing Molecule	Electrochemical Technique Used	Fabrication Strategy	Fabrication Steps	Paper Type	LDR	LOD	Real Sample	References
1	HPV	SWV	Combining G-PANI modified electrode with an AQ-labeled acpcPNA probe to create DNA biosensor.	5	Filter paper (Whatman No. 1)	10–200 nM	2.3 nM	NR	[77]
2	K-562 cell	DPV	Fabrication of folic acid-functionalized Au-paper working electrode on origami electrochemical device.	4	Cellulose paper	1.0 × 10^2^–1.0 × 10^6^ cells/mL	31 cells/mL	Human serum	[94]
3	*Staphylococcus aureus*	DPV	Ab-SWCNT bioconjugates immobilized on working electrode.	5	Chromatography paper (Whatman)	0–10^7^ CFU/mL	13 CFU/mL	Food samples	[95]

HPV—Human papilloma virus, SWV—Square wave voltammetery, G-PANI—Graphene-polyaniline, AQ-labeled acpcPNA—Anthraquinone-labeled pyrrolidinyl peptide nucleic acid, DPV—Differential pulse voltammetry, SWV—Square wave voltammetry, Ab—Antibody, SWCNT—Single-walled carbon nano tube, NR—Not reported.

## Data Availability

Not applicable.
